# Kinetic Intricacies of the Light Emission and Antiradical Influence of Exogenous Bioantioxidants Transformation Products in the Chemiluminescence Bioantioxidant Assay

**DOI:** 10.3390/ijms24108486

**Published:** 2023-05-09

**Authors:** Vladimir V. Naumov, Aleksei V. Trofimov, Galina F. Fedorova, Olga I. Yablonskaya, Rostislav F. Vasil’ev

**Affiliations:** 1 Emanuel Institute of Biochemical Physics, Russian Academy of Sciences, Ul. Kosygina 4, 119334 Moscow, Russia; my9name@mail.ru (V.V.N.); sellerey@mail.ru (G.F.F.); olga.yablonsky@gmail.com (O.I.Y.); rfv-28@mail.ru (R.F.V.); 2 Moscow Institute of Physics and Technology (National Research University), Institutskii per. 9, 141701 Dolgoprudny, Russia

**Keywords:** bioantioxidants, bioluminescence modeling, chemiluminescence, excited-state generation, kinetic analysis, reactive oxygen species

## Abstract

The subject matter of the reported work refers to studying the interactions followed by the excited-state generation, which are chemical models of oxidative processes leading to a weak light emission emerging from living cells, and to explore the possibilities of using them as tools for evaluating the activity of oxygen-metabolism modulators, most prominently, natural bioantioxidants of biomedical value in particular. Methodologically, major attention is paid to analyzing the shapes of the time profiles of the light emission derived from a model sensory system in the presence of lipid samples of vegetable and animal (fish) origin rich in bioantioxidants. As a result, a modified reaction mechanism involving 12 elementary steps is proposed to rationalize the light-emission kinetics in the presence of natural bioantioxidants. We conclude that free radicals formed from bioantioxidants and their dimerization products contribute significantly to the general antiradical activity of lipid samples, which should be taken into account in developing efficient bioantioxidant assays for biomedical applications and while establishing the mechanisms of bioantioxidant effects on metabolic processes in vivo.

## 1. Introduction

It has long been known that numerous life forms produce light ranging from the ultra-weak spontaneous glow of cells and tissues to the bright bioluminescence of fireflies and a variety of marine organisms [1,2,3,4,5]. Thus, understanding the mechanisms of biological luminescence in all its manifestations and finding ways to use this property of living nature are fundamental tasks of modern science, which are dictated by practice [1,2,3,4]. In this regard, it is noteworthy that “live” photons are born in oxidative interactions [1,2,3,4,5,6,7], i.e., in processes without which the vital activity of organisms would be completely unthinkable, and the precursors of excited particles, light sources, are the reaction products of biomolecules with oxygen. Hence, oxygen, light and life are in a close relationship, and its study is necessary both for the elucidating the function of biological systems and for diagnosing the development of oxidative stress, which is the prominent cause of cellular dysfunctions and a universal generator of pathologies of different origin [7]. It should also be noted that diverse exogenous compounds, both benign and harmful ones, influence oxygen metabolism, thereby affecting the generation of light in vivo [7] and predetermining the development of bio- and chemiluminescence techniques for medical diagnostics, pharmacological research and toxicology [7]. However, actually, in pursuing these aims, the most suitable way of designing the appropriate luminescence sensory systems consists in harnessing simple and well-controlled chemical reactions, mimicking the main features of light generation in the “luminous” bioprocesses. Such experimental tools are simpler to handle and do not require easy perishable biological materials [2,4,5,6,7,8,9,10]. The scope of the present work pertains to studying the interactions leading to light emission, which, in fact, constitute the chemical models of oxidative processes accounting for the luminescence of living cells [5,6,7,8,9], and examining the possibilities of their use as sensory systems for assessing the antiradical activity of natural chain-breaking bioantioxidants.

Bioantioxidant ingredients of food, medicines and other products are most frequently considered in terms of the protective role they play in living organisms that consume these constituents. However, it is noteworthy that overloading with exogenous antioxidants (including antioxidants in environmental pollution [11] and in cigarette smoke [12,13]) also represents a certain challenge for toxicology and ecotoxicology in the elucidation of the mechanisms of toxic effects.

As sources of bioantioxidants in the present work, natural lipid samples were used. Natural lipids, structural components of cell membranes, are protected from oxidative degradation, most prominently by bioantioxidant molecules abundant in living cells [8]. Among the variety of experimental tools to examine the antiradical activity of lipid materials, kinetic chemiluminescence methods are the most facile and efficient [8,9].

The choice of chemiluminescence-based and related approaches depends on the nature of the analytes and their chemical activity. For example, when charge separation or electron-transfer processes may take place, electrochemiluminescence methods [14] and other complementary approaches [15] are the most appropriate. In the context of the present work, it should be emphasized that chemiluminescence methodologies are particularly advantageous for the online monitoring of the reaction kinetics [16], which, in turn, is of prime importance for acquiring pertinent mechanistic insights.

**Scheme 1:**
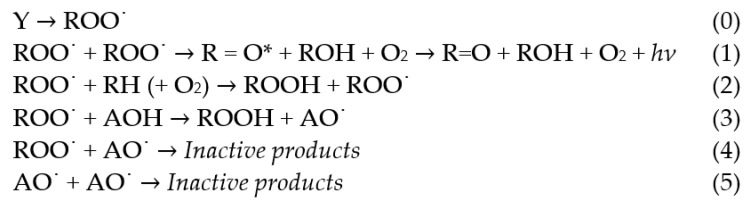
Hydrocarbon (RH) oxidation and chemiluminescence mechanism in the presence of bioantioxidant (AOH).

In a chemiluminescence sensory system that allows measuring the level of free peroxide radicals (ROO˙), the disproportionation reaction of which (reaction (1), Scheme 1) is accompanied by the light emission, the oxidation of a model hydrocarbon substrate (RH) is used for the controlled ROO˙ generation. To acquire the pertinent quantitative characteristics from experimental data on the light intensity as a function of a bioantioxidant (AOH) concentration, kinetic calculations are usually made on the basis of Scheme 1 (reactions (0)–(5)) [8,9].

The use of such a model chemiluminescence process consists of measuring the intensity of the light emission (derived from an electronically excited product, ketone R = O*) as the function of [AOH].

As the pertinent alternative to such an approach, bioantioxidant assay utilizing luminol chemiluminescence is noteworthy [17].

The ability of chain-breaking bioantioxidants to inhibit oxidation processes is governed by their propensity to interact with peroxide radicals ROO˙ according to reaction (3), the rate constants of which are the characteristics of the antiradical activity of bioantioxidants [8,9]. Typical kinetic curves of the chemiluminescence intensity (*J*) in the case of the reaction mechanism exhibited in Scheme 1 bear a symmetrical S-shape with a pronounced induction period when the light emission is practically absent due to a complete scavenging of ROO˙ by AOH, which is followed by a recovery of the light-emission intensity on a gradual consumption of AOH in the oxidation process [8,9]. The reaction rate constant *k*_3_ is then estimated based on its proportionality to the maximal slope, (d*J*/dt)_max_, at the inflection point on the chemiluminescence-intensity time profile, *J*(t), which should remain constant at different antioxidant concentrations [8,9].

However, the question arises, how would the light-emission kinetics differ from the ideal case (Scheme 1) if by-products with antiradical activity are formed in the oxidation process? For instance, it is known that natural bioantioxidants are capable of forming dimers and other products containing hydroxyl groups, the presence of which is typical for radical scavengers [18,19,20,21]. This issue is of prime importance for the further design of efficient and reliable bioantioxidant sensory systems, most prominently for biomedical applications (e.g., for assaying the intricacies of bioantioxidant activity of diverse biological samples, drugs and their stabilizers, as well as environmental pollutants). In the present work, we examined the antiradical activity of natural lipids to establish a possible mechanism for the influence of bioantioxidant products on the chemiluminescence kinetic and to expand model concepts of their action on the oxidation processes in order to improve the reliability of bioantioxidant assays.

## 2. Results

**Figure 1:**
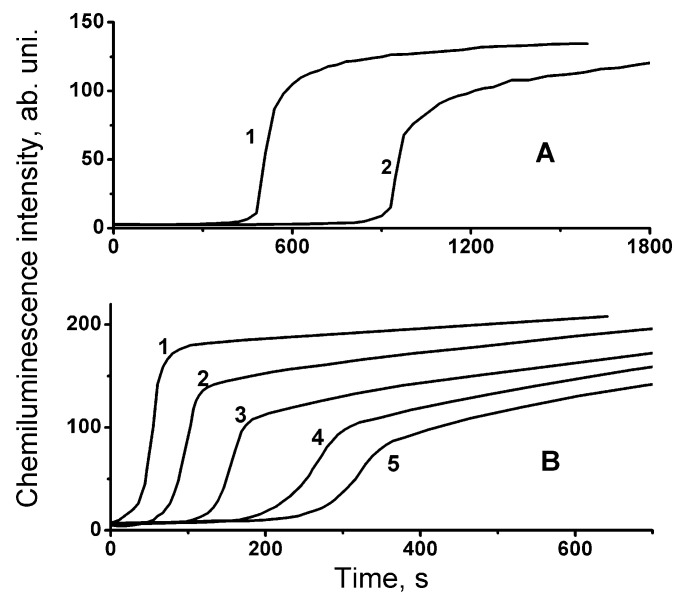
Chemiluminescence kinetics in the presence of lipid-born bioantioxidants: (**A**) initiated by AIBN (8 × 10^−3^ M) oxidation of sunflower oil in chlorobenzene [2% (1), 4% (2) of the volume] at 50 °C, (**B**) when lipids from saffron cod (*Eleginus gracilis*) are introduced into the chemiluminescent ‘cocktail’ of oxidizing ethylbenzene at concentration of 0.68 (1), 1.4 (2), 2.7 (3), 4.1 (4), 5.4 (5) g/L and at the initiation rate of 4.4 × 10^−8^ Ms^−1^ and 50 °C.

**Figure 2:**
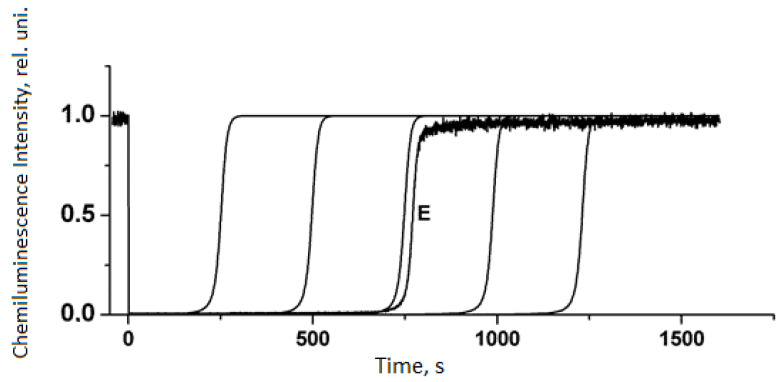
Kinetic curves of the chemiluminescence intensity manifesting the antiradical effect of antioxidants in the chlorobenzene solution of ethylbenzene (1.86 M) upon its oxidation by the ambient oxygen at the initiation rate of 2.43 × 10^−8^ Ms^−1^. Experimental curve E refers to the action of α-tocopherol at the concentration of 9.06 µM, while the other curves are the results of computer modeling in a framework of Scheme 1 at initial antioxidant concentrations of 3, 6, 9.06, 12, 15 µM, correspondingly.

Chemiluminescence kinetic curves measured in the presence of lipids are exhibited in Figure 1. Inspection of the acquired intensity time profiles reveals the following qualitative features of the chemiluminescence kinetics. The exhibited curves are S-shaped with a pronounced induction period. However, the shapes of these curves are asymmetric, in contrast to the curves known for synthetic inhibitors of the free-radical oxidation [8,9], whose symmetry is illustrated by the chemiluminescence time profiles (Figure 2) obtained by computer modeling using COPASI software package [22,23] based on the mechanism represented by Scheme 1. In this context, characteristic differences are (i) the presence of a gently sloping area at the end of the “dark” induction period and (ii) the decrease in the maximal slope, (d*J*/dt)_max_, at the inflection point on the chemiluminescence time profile, *J*(t), as the initial concentration of an antioxidant increases (Figure 1).

For the chemiluminescence process, which obeys the mechanism exhibited in Scheme 1, the value of the maximal slope, (d*J*/dt)_max_, at the inflection point on the chemiluminescence-intensity time profile should remain constant at different antioxidant concentrations [8,9]. Verification of such a contention was carried out in the present work using computer mathematical modeling [22]. For these computations, we used the following basic set of the rate constants (their numbering refers to that of the reaction steps in Scheme 1): *k*_1_ = 1 × 10^7^, *k*_2_ = 1.7, *k*_3_ = 3.6 × 10^6^, *k*_4_ = 1 × 10^8^, *k*_5_ = 1 × 10^3^ M^−1^s^−1^. These values have been chosen in accordance with the rate constants of similar reactions available in the literature [5,6,20,23,24,25]. The computational results confirm the constancy of the (d*J*/dt)_max_ value, which is inconsistent with the experimental finding. Indeed, a clear decrease in the slope, (d*J*/dt)_max_, of the kinetic curves, *J*(t), was observed with the increase in the initial concentration of bioantioxidants (Figure 1). Consequently, Scheme 1 is not complete and needs to be mechanistically extended.

**Scheme 2:**
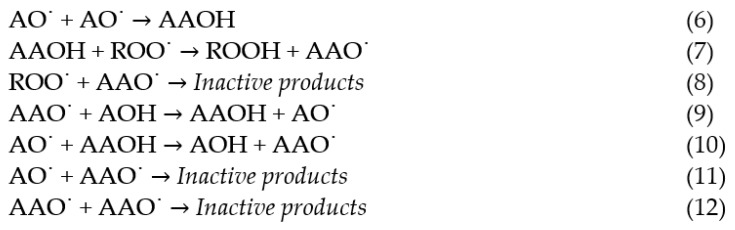
Extended oxidation mechanism upon the bioantioxidant transformation.

We assumed that the chemiluminescence kinetics were influenced by the products of the transformation of bioantioxidants capable of reacting with free peroxide radicals, ROO˙. Thus, the additional elementary reactions, which are exhibited in Scheme 2, need to be added to the reaction mechanism; the latter constitutes the extension of Scheme 1. These additional reaction steps refer to the formation of the AAOH dimer (reaction (6)) and its interaction with the peroxide radicals, ROO˙, to form the radicals AOO˙ (reaction (7)). Furthermore, the interactions of the mentioned species with the other components of the reaction system (reactions (8)–(12)) were added to the extended reaction sequence, on the basis of which further computer modeling was carried out.

**Figure 3:**
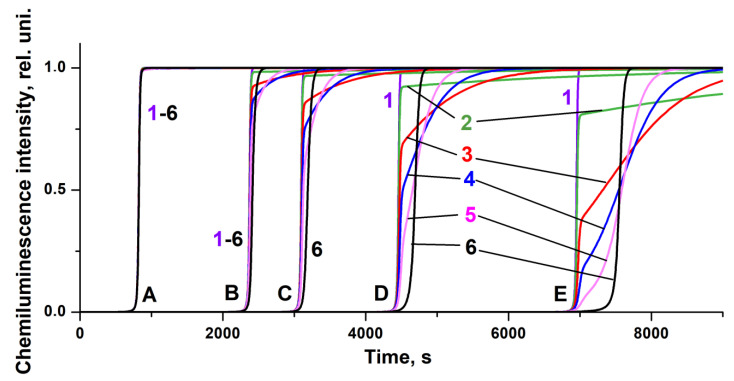
Computed chemiluminescence kinetic curves at the *k*_7_ values equal to 0 (1), 1 × 10^4^ (2), 5 × 10^4^ (3), 1 × 10^5^ (4), 2 × 10^5^ (5), 1 × 10^6^ (6) M^−1^s^−1^. The values of the initial antioxidant concentration are 1 × 10^−5^ (A), 3 × 10^−5^ (B), 4 × 10^−5^ (C), 6 × 10^−5^ (D), 1 × 10^−4^ (E) M; the reaction initiation rate is 2.43 × 10^−8^ M^−1^s^−1^.

In such a reaction sequence, steps 7 and 8 are the steps that contribute additionally to the light-emission quenching since they constitute extra channels for scavenging peroxide radicals. As a result of the computer simulation based on this extended reaction mechanism, it was found that the shape of the chemiluminescence time profiles strongly depends on the reactivity of the formed AAOH dimers towards peroxide radical ROO˙ (reaction (7) with the rate constant *k*_7_). Figure 3 exhibits five sets (A–E) of the kinetic curves obtained at different initial antioxidant concentrations and six types of curves corresponding to different values of the constant *k*_7_. For the rate constants of the other elementary reactions (Scheme 2) added to the basic set (Scheme 1), the following input values were taken: *k*_6_ = 10^3^, *k*_8_ = 10^8^, *k*_9_ = 10^4^, *k*_10_ = *k*_11_ = *k*_12_ = 10^3^ M^−1^s^−1^, based on the available literature data [5,6,20,23,24,25].

It is noteworthy that although the interaction of peroxide radicals with AOH constitutes the major way of the ROO˙ scavenging (leading to light quenching and manifested by the high *k*_3_ value), effectively competing with the ROO˙ disproportionation (leading to light emission), the overall chemiluminescence kinetics is rather sensitive to the reaction with AAOH. Thus, the increase of *k*_7_ in the range of 10^4^ to 10^5^ M^−1^s^−1^ reduces the maximum slope, (d*J*/dt)_max_, of the kinetic curves, *J*(t), and leads to the appearance of a relatively gently sloping area on the chemiluminescence time profiles at the end of the induction period (Figure 3), indicating the presence of an antioxidant product at the end of the process with relatively low antiradical activity, which suppresses the oxidation (and chemiluminescence) only partially. The presence of such gently sloping parts of the chemiluminescence time profiles can also be observed on the experimental light-intensity curves manifesting the effects of lipid antioxidants (Figure 1), as well as on the chemiluminescence kinetic curve in the presence of α-tocopherol (Figure 2).

In general, the importance of the influence of secondary antioxidant products may also explain the different antiradical activity assessed for some chain-breaking antioxidants in various in vitro assays.

The correspondence between the experimental and the computed chemiluminescence kinetic curves confirms the adequacy of the model kinetic pattern obtained on the basis of the extended mechanism involving 12 elementary reactions (Scheme 1 and Scheme 2). A further increase in the constant *k*_7_ to 10^6^ M^−1^s^−1^ leads to an increase in the induction period (Figure 3). At the same time, the slope, (d*J*/dt)_max_, of the curves of type 6 is less than that of the curves of type 1, which corresponds to the difference in the rate constants *k*_7_ and *k*_3_, indicating that the end of the induction period on curves of 6 type corresponds to the predominant consumption of the AAOH product with lower antiradical activity (rate constant *k*_7_) compared to the activity of the original antioxidant AOH (rate constant *k*_3_).

**Figure 4:**
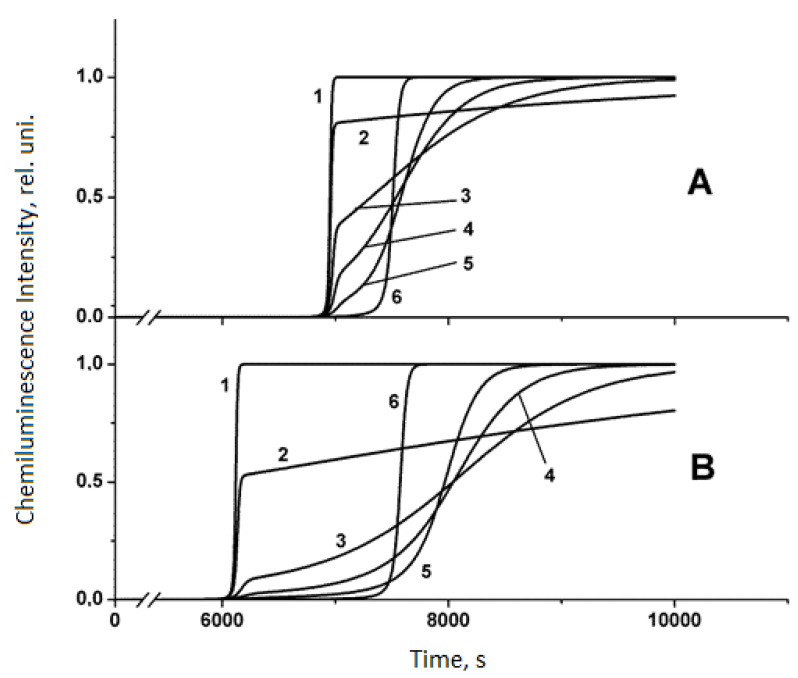
Influence of the rate constant of reaction (6) (Scheme 2) on the kinetics of chemiluminescence emission: *k*_6_ = 10^3^ (**A**), *k*_6_ = 10^4^ (**B**) M^−1^s^−1^. In other designations, the values of *k*_7_ and the reaction initiation rate are the same as in the case of Figure 3.

Reaction (6) of free radicals AO˙ formed from antioxidant molecules is the key to the formation of the active AAOH species capable of interacting with peroxide radicals, ROO˙, according to reaction (7) and thus affecting the kinetics of the free-radical oxidation process and the character of the chemiluminescence emission. This reaction competes with reaction (5), the products of which are non-reactive. Figure 4 shows the two sets (A and B) of the model chemiluminescence kinetic curves obtained at the same antioxidant concentration (10^−5^ M) but at different values of the rate constant of reaction (6): *k*_6_ = 10^3^ M^−1^s^−1^ (A) and *k*_6_ = 10^4^ M^−1^s^−1^(B). The mechanistic and kinetic implications of these computational results will be accordingly addressed in the Discussion section.

## 3. Discussion

The results of the present study revealed that the shape of the computed chemiluminescence kinetic curves exhibited in Figure 3 and Figure 4 corresponds to the shape of the experimental curves in Figure 1 and Figure 2, manifesting both the decrease in the value of the maximal slope ((d*J*/dt)_max_) of the intensity curve, *J*(t), with the increase in the initial concentration of the reaction inhibitor and the presence of the gently sloping area on emission-intensity time profiles. The efficiency and the nature of the inhibitory activity largely depend both on the possibility of the formation of antioxidant products in reaction (6) (Scheme 2) and on their antiradical activity expressed by the rate constant *k*_7_ of reaction (7). The extended reaction mechanism (Scheme 1 and Scheme 2) involves 12 elementary steps, including the stages of formation of dimeric antioxidant products and their participation in the chain oxidation process, which adequately accounts for all the features of the experimentally observed chemiluminescence kinetics in the presence of natural bioantioxidants, which expands the possibilities of examining and monitoring the bioantioxidant activity.

The intricacies of the chemiluminescence kinetics derived from newly considered elementary reaction steps are particularly noteworthy. First of all, a comparison of the “equikinetic” chemiluminescence time profiles (i.e., the ones which pertain to the same values of *k*_7_) reveals that the effect of reaction (6) on the chemiluminescence emission is ambiguous. Thus, we have noticed that at small *k*_7_ values (curves 1 and 2 in Figure 3 and Figure 4), the replacement of *k*_6_ = 10^3^ by *k*_6_ = 10^4^ M^−1^s^−1^ (transition from A to B in Figure 4) reduces the induction period since the increase in *k*_6_ lowers the concentration of free radicals AO˙ and, hence, the probability of their interaction with peroxide radicals, while the AAOH dimer formed by reaction (6) is insufficiently active due to low *k*_7_. Conversely, at high values of *k*_7_ = 10^5^ to 10^6^ M^−1^s^−1^, a comparison of the “equikinetic” light-intensity curves (4–6 in A and B, Figure 4) reveals the higher antioxidant activity in case B. This can be rationalized by the fact that, due to the enhancement of the activity of the AAOH dimer (an increase of *k*_7_ by the order of magnitude), the contribution of reactions (7) and (8) (removing peroxide radicals) increases, which leads to the inhibition of the chain oxidation process.

The major advantage of using chemiluminescence sensory systems instead of their bioluminescence prototypes is that the former is much easier to handle. However, as the present study shows, even in such a simplified version of the light-signal generation, mechanistic intricacies may significantly affect the interpretation of the experimental results while analyzing the activity of biological samples, and the possible influence of the potential by-products needs to be thoroughly considered. Clearly, the formation of the considered herein active by-products needs to be taken into account both in developing reliable bioantioxidant assays and while establishing the mechanisms of bioantioxidant effects on metabolic processes in vivo.

## 4. Materials and Methods

Measurements of the chemiluminescence emission were conducted using the Hamamatsu photosensor unit H7467 supplemented with the RS-232C interface as previously described [8,9,23].

The chemiluminescence sensory system consisted of a hydrocarbon ‘cocktail’, which contained a chlorobenzene solution of ethylbenzene (RH) subject to oxidation initiated by free radicals derived from 2,2′-azobisisobutyronitrile (AIBN) upon its thermal decomposition [8,9,23]. As the bioantioxidant standard, α-tocopherol was applied. All the chemicals were purchased from standard suppliers and were purified according to the published procedures [26].

Research samples of the sunflower oil have been generously donated by Professor Vessela Kancheva (Institute of Organic Chemistry with the Center of Phytochemistry, Bulgarian Academy of Sciences). Fish-derived lipids were extracted from the muscle tissue of saffron cod (*Eleginus gracilis*) with a binary solvent mixture according to the standard methodology [27]. Computer mathematical modeling of the kinetics of the oxidative process and chemiluminescence emission upon bioantioxidants action was carried out using a COPASI software package [22], as was done in our recent study [23]. The rate constants available from the literature [5,6,20,23,24,25] were recalculated to the temperature of 50 °C. In the course of computer modeling, these values were altered for up to two orders of magnitude. The concentration of oxygen for these computations has been taken as 0.00236 M according to its solubility in chlorobenzene [28].

## 5. Conclusions

The present study reveals the new characteristic features of the light-intensity time profiles upon the addition of natural lipid samples into the chemiluminescence sensory system based on the free-radical chain oxidation of a model hydrocarbon substrate: (i) the maximal slope, (d*J*/dt)_max_, of the chemiluminescence kinetic curve, *J*(t), decreases with the increase in the initial concentration of the lipid-born bioantioxidant analyte, and (ii) the gently sloping area of the light-emission kinetics is observed at the end of the induction period of the oxidation process.

A reaction mechanism of 12 elementary steps is proposed, which accounts for the observed chemiluminescence kinetics in the presence of lipid-born antioxidants.

Free radicals formed from bioantioxidants and their dimerization products contribute significantly to the general antiradical efficiency of lipid samples, which should be taken into account in developing the efficient bioantioxidant assays for biomedical applications and in considering the mechanisms of altering the natural metabolic equilibrium upon consumption of exogenous bioantioxidants.

Clearly, challenges in chemiluminescence studies of biological samples with antioxidant activity are not limited to the kinetic intricacies considered herein. First of all, for further development of effective and reliable chemiluminescence bioantioxidant assays, the follow-up analysis of the ways and conditions of possible conversion of antioxidant properties into prooxidant ones needs to be carried out. To address this problem, further elaboration of experimental approaches, most prominently for studying the kinetics and stoichiometry of the bioantioxidants’ action as a function of their concentration, is required along with the subsequent development of the computer mathematical modeling of oxidative processes in the presence of bioantioxidants.
